# Analysis of GWAS-Derived Schizophrenia Genes for Links to Ischemia-Hypoxia Response of the Brain

**DOI:** 10.3389/fpsyt.2020.00393

**Published:** 2020-05-12

**Authors:** Rainald Schmidt-Kastner, Sinan Guloksuz, Thomas Kietzmann, Jim van Os, Bart P. F. Rutten

**Affiliations:** ^1^Integrated Medical Science Department, C.E. Schmidt College of Medicine, Florida Atlantic University (FAU), Boca Raton, FL, United States; ^2^Department of Psychiatry and Neuropsychology, School for Mental Health and Neuroscience, Maastricht University Medical Centre, Maastricht, Netherlands; ^3^Department of Psychiatry, Yale School of Medicine, New Haven, CT, United States; ^4^Faculty of Biochemistry and Molecular Medicine and Biocenter Oulu, University of Oulu, Oulu, Finland; ^5^Department of Psychiatry, Brain Center Rudolf Magnus, University Medical Center Utrecht, Utrecht University, Utrecht, Netherlands; ^6^Department of Psychosis Studies, Institute of Psychiatry, King’s College London, King’s Health Partners, London, United Kingdom

**Keywords:** schizophrenia, gene-environment (G-E) interaction, obstetric complications, ischemia, hypoxia, HIF, gene expression, synapse

## Abstract

Obstetric complications (OCs) can induce major adverse conditions for early brain development and predispose to mental disorders, including schizophrenia (SCZ). We previously hypothesized that SCZ candidate genes respond to ischemia-hypoxia as part of OCs which impacts neurodevelopment. We here tested for an overlap between SCZ genes from genome-wide association study (GWAS) (n=458 genes from 145 loci of the most recent GWAS dataset in SCZ) and gene sets for ischemia-hypoxia response. Subsets of SCZ genes were related to (a) mutation-intolerant genes (LoF database), (b) role in monogenic disorders of the nervous system (OMIM, manual annotations), and (c) synaptic function (SynGO). Ischemia-hypoxia response genes of the brain (IHR genes, n=1,629), a gene set from RNAseq in focal brain ischemia (BH, n=2,449) and genes from HypoxiaDB (HDB, n=2,289) were overlapped with the subset of SCZ genes and tested for enrichment with Chi-square tests (p < 0.017). The SCZ GWAS dataset was enriched for LoF (n=112; p=0.0001), and the LoF subset was enriched for IHR genes (n=25; p=0.0002), BH genes (n=35; p=0.0001), and HDB genes (n=23; p=0.0005). N=96 genes of the SCZ GWAS dataset (21%) could be linked to a monogenic disorder of the nervous system whereby IHR genes (n=19, p=0.008) and BH genes (n=23; p=0.002) were found enriched. N=46 synaptic genes were found in the SCZ GWAS gene set (p=0.0095) whereby enrichments for IHR genes (n=20; p=0.0001) and BH genes (n=13; p=0.0064) were found. In parallel, detailed annotations of SCZ genes for a role of the hypoxia-inducible factors (HIFs) identified n=33 genes of high interest. Genes from SCZ GWAS were enriched for mutation-intolerant genes which in turn were strongly enriched for three sets of genes for the ischemia-hypoxia response that may be invoked by OCs. A subset of one fifth of SCZ genes has established roles in monogenic disorders of the nervous system which was enriched for two gene sets related to ischemia-hypoxia. SCZ genes related to synaptic functions were also related to ischemia-hypoxia. Variants of SCZ genes interacting with ischemia-hypoxia provide a specific starting point for functional and genomic studies related to OCs.

## Introduction

A core concept of contemporary psychiatric research is that multiple genes interact with environmental factors to increase the risk of psychosis spectrum disorder, including schizophrenia (1–4). Obstetric complications (OCs) are established risk factors for schizophrenia (5–7) while it is generally thought that perturbations of oxygen and substrate delivery (i.e., ischemia-hypoxia) during OCs affect the developing brain and impair neuronal development during critical phases (8, 9). Immune mechanisms and neuroinflammation are also considered as major prenatal risk factors for schizophrenia (10), and inflammation typically invokes hypoxic challenges (11). Furthermore, evidence for links between vascular factors, including angiogenesis, and schizophrenia has been reported (12–14).

We previously hypothesized that candidate genes for schizophrenia interact with ischemia-hypoxia during OCs (9). To explore this hypothesis, we used our “ischemia-hypoxia response” (IHR) database that combined information for changes at the mRNA level after brain ischemia-hypoxia from multiple experimental studies (15, 16). Candidate genes for schizophrenia were then annotated using the IHR gene database (9, 17). Thereby, the assumptions were made that those genes observed experimentally in the adult brain are likely expressed in the developing brain, and that genes transcribed in mice or rats are also expressed in the human brain. The concept was extended to the expression or function in vascular cells, because ischemia-hypoxia will immediately invoke vascular responses and genetic mechanisms affecting vessels may cause ischemia-hypoxia. The hypothesis was that genetic variants of schizophrenia genes annotated as IHR genes and/or vascular genes may alter responsiveness to ischemia-hypoxia during OCs. In fact, our hypothesis has received support from studies where the interaction between SNPs for selected candidate genes were linked to IHR genes and OCs in relation to schizophrenia risk (18–20).

The initial analysis (9) was based on a selection from SzGene which collected more than 700 candidate genes for schizophrenia (21). A bias within SzGene toward genes of major interest for brain damage and neurodegeneration would have inflated the importance of IHR genes. Genome-wide studies such as GWAS are now considered as standard for the hypothesis-free analysis of polygenic disorders such as schizophrenia. Therefore, it was timely to examine the genes in loci defined by GWAS in schizophrenia (22, 23) in relation to ischemia-hypoxia. To do this, we linked the schizophrenia GWAS dataset to ischemia-hypoxia using the following approaches to form relevant subsets. 1) A large set of mutation-intolerant genes (LoF) was recently described (24), and it was reported that common schizophrenia alleles in GWAS are enriched for mutation-intolerant genes (23). It was hypothesized that IHR genes should be also enriched in datasets of LoF if they are essential for the response to ischemic-hypoxic challenges. Then, tests were performed for an overlap between schizophrenia genes from GWAS, mutation-intolerant schizophrenia genes, and IHR genes. 2) Schizophrenia genes from GWAS were annotated for known monogenic disorders of the nervous system that affect development and function, and then tested for overlap with IHR genes. 3) Since schizophrenia genes emerging from genomic studies are closely related to synaptic functions (25, 26), a novel database of synaptic proteins [SynGO; (27)] was used to define IHR genes related to synaptic functions and tested for the overlap with schizophrenia genes. 4) Studies were supplemented by analyzing a recently proposed set of 104 schizophrenia candidate genes from the original 108-loci study using a complex combination of parameters (28). 5) To test for a more general involvement of neurodevelopmental disease mechanisms, a gene set related to developmental delay and autism spectrum disorder (29) was tested for enrichment as well. To further explore a to link to ischemia-hypoxia, a gene set from a recent RNAseq study in focal brain ischemia (30) and the HDB database (31) were employed.

## Materials and Methods

**Figure 1:**
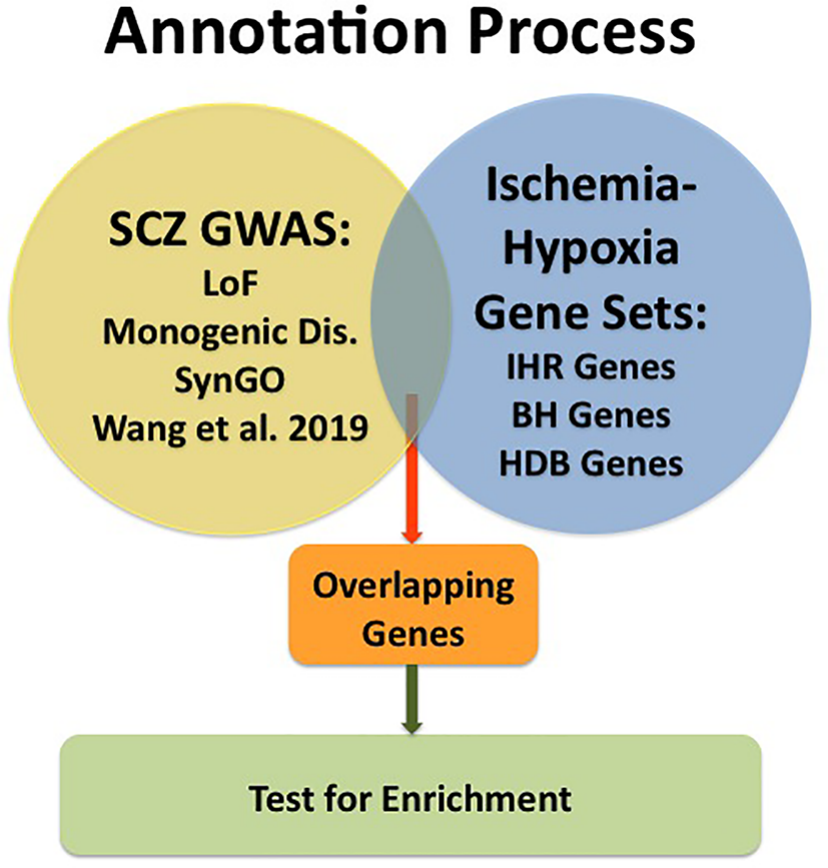
Graphic presentation of the annotation process. GWAS data for schizophrenia (SCZ GWAS) were taken from Pardinas et al. [ref. (23)] and subsets of genes were formed for Loss of Function [LoF; ref. (24)], for Monogenic Disorders (Monogenic Dis.; described under Materials and Methods), for synaptic genes collected in SynGO [ref. (27)] and for a subset described by Wang et al., 2019 [ref.(28)]. Gene sets related to Ischemia-Hypoxia were taken from our database named “ischemia-hypoxia response genes” [IHR Genes; refs. (16, 17)], from the RNAseq study of Bhattarai et al., 2019 [BH Genes; ref. (30)] and from the public database named HypoxiaDB [HDB Genes; ref. (31)]. Overlapping genes were determined, and enrichment tested using Chi-square tests.

An overview of the analysis is provided in **Figure 1**.

### Databases for IHR

The database of “ischemia-hypoxia response” (IHR) genes contains manually curated genes (n=1,629) from n=24 microarray studies in experimental brain ischemia-hypoxia. This cumulative database was first described in a study of retinal genes (15) and an update has been reported (16). Most of the genes are upregulated (higher mRNA levels) in experimental studies. The definition of “response” relates to regulation of gene expression but also includes other mechanisms, i.e. mRNA splicing, stability, and degradation. The curation included alignment of mouse or rat gene symbols with human gene symbols; this was initially achieved by fusing the IHR gene list with a human gene list (32) and then by resolving conflicts by detailed searches in Entrez Gene and HomoloGene. BLAST searches for the microarray probe were used in some cases. We estimate that the combined IHR genes (n=1,629) represent 9% of genes potentially expressed during brain development; this was based on a conservative estimate of n=20k for the total number of protein-coding genes in the genome, the report for >90% of genes being expressed in the developing human brain (33), and on the assumption of genome-wide coverage of the microarrays. Then enrichment in a specific schizophrenia gene set was estimated by comparing the observed overlap with the randomly expected overlap set at 9%.

A recent study of focal brain ischemia in mice has used RNAseq analysis at three different time points (30); these data were combined as “BH-genes,” n=2,449 (14%). Since this dataset uses the sensitive RNAseq method and a single experimental model, it was considered as a replication test for the microarray-based, cumulative dataset of IHR genes.

HDB is a database related to proteomic findings for hypoxia in general with n=2,289 entries [12%; ref. (31)]. This database is composed of findings made at the protein level across multiple organs (31) and therefore is independent of expression in the brain. For convenience, this dataset was named “HDB genes” in this analysis.

### Selection of Subsets of GWAS-Based Schizophrenia Genes

N=458 genes from 145 loci of the recent GWAS dataset in schizophrenia were retrieved (23). Subsets of genes were then selected using specific annotations.

The database of loss of function (LoF) (24) was used to retrieve mutation-intolerant genes (n=3,230).

A manual annotation of GWAS-based schizophrenia genes (n=458 genes from 145 loci) was carried out for monogenic disorders that are known to affect development and function of the nervous system. Gene symbols were searched in OMIM™ (https://www.omim.org), the DDD-database (34), or DisGeNET (35); this was supplemented by abstract searches in PubMed. Genes were included when a single gene mutation has been described to cause a neurodevelopmental disorder, intellectual disability, neurological disease, or diseases of the retina and hearing nerve.

SynGO (27) was used to define synaptic proteins (n=1,112) among GWAS-based schizophrenia genes.

A subset of schizophrenia genes from GWAS analysis was defined in a recent genomic study that used a Bayesian framework to integrate multiomics data and gene networks [n=104 genes; ref. (28)].

### Neurodevelopmental Disorders

To test whether the overlap with ischemia-hypoxia can be extended to neurodevelopmental disorders, a collection of genes defined by *de novo* mutations in developmental delay and autism spectrum disorder (n=253) was adopted, named DD/ASD (29).

### Exploration of Hypoxia-Inducible Factors

Hypoxia-inducible factors (HIFs) are major transcriptional regulators of the hypoxia response (36, 37), including the brain (38). For annotation of schizophrenia genes, information for HIF-targets and HIF-regulators was compiled from multiple databases and literature.

### Analyses

Subsets of schizophrenia genes (as defined above) and DD/ASD were overlapped with the three gene sets for response to ischemia-hypoxia. Testing for enrichment was performed by comparing the observed number of shared genes to the randomly expected number of genes using Chi-square tests (p < 0.017 was considered as statistically significant, given the three comparisons). As additional test for a link to synaptic genes, the IHR gene dataset was subjected to annotation by DAVID Bioinformatics (https://david.ncifcrf.gov).

## Results

The overall goal of this study was to uncover links between experimentally defined genes of the IHR (as collected in three databases, i.e., IHR, BH, and HDB) and genes from schizophrenia GWAS. Following the analysis of the total gene set, four subsets of genes from schizophrenia GWAS were formed and tested for enrichment. For comparison, a gene set for neurodevelopmental disorders was subjected to the same analysis.

### Total Gene Set From Schizophrenia GWAS

Some genes well-known from experimental studies of brain ischemia-hypoxia stood out following the annotation of the total gene set from schizophrenia GWAS for IHR genes (**Table 1**). N=51 IHR genes, n= 59 BH genes, and n=48 HDB genes were overlapping with schizophrenia genes; however, none of these individual datasets showed enrichment in the total schizophrenia GWAS dataset. Therefore, subsets of the GWAS datasets were formed using criteria specified above and tested for enrichment with gene sets related to ischemia-hypoxia.

**Table 1:** Selected schizophrenia genes derived from genome-wide association study (GWAS) matching well-recognized ischemia-hypoxia response (IHR) genes of the brain.

Gene symbol	Official full name	Main biological function
AKT3	AKT serine/threonine kinase 3	Serine/threonine protein kinase, growth factor signaling
ATP2A2	ATPase sarcoplasmic/endoplasmic reticulum Ca2+ transporting 2	Intracellular calcium pump associated with ER
BNIP3L	BCL2 interacting protein 3 like	Pro-apoptotic factor within the Bcl-2 family
CACNA1C	Calcium voltage-gated channel subunit alpha1 C	Alpha-1 subunit of a voltage-dependent calcium channel
CACNB2	Calcium voltage-gated channel auxiliary subunit beta 2	Subunit of a voltage-dependent calcium channel protein
CLU	Clusterin	Secreted chaperone
FGFR1	Fibroblast growth factor receptor 1	Growth factor signaling
FURIN	Furin, paired basic amino acid cleaving enzyme	Subtilisin-like proprotein convertase
HSPA9	Heat shock protein family A (Hsp70) member 9	Heat shock protein 70 gene family, primarily mitochondrial
HSPD1	Heat shock protein family D (Hsp60) member 1	Member of the chaperonin family, mitochondrial, HSP60
HSPE1	Heat shock protein family E (Hsp10) member 1	Major heat shock protein, HSP10
INA	Internexin neuronal intermediate filament protein alpha	Intermediate filament protein, in axonal cytoskeleton
MDK	Midkine	Secreted growth factor
MEF2C	Myocyte enhancer factor 2C	Transcription enhancer, trans-activating, DNA binding activities
NCAN	Neurocan	Modulation of cell adhesion and migration
NGEF	Neuronal guanine nucleotide exchange factor	Guanyl-nucleotide exchange factor activity
NRGN	Neurogranin	Postsynaptic protein kinase, binding calmodulin
OPCML	Opioid binding protein/cell adhesion molecule like	Cell adhesion, accessory role in opioid receptor function
PTK2B	Protein tyrosine kinase 2 beta	Protein tyrosine kinase, regulation of ion channels
RANGAP1	Ran GTPase activating protein 1	Regulation of nuclear transport, GTP-binding and exchange
RELA	RELA proto-oncogene, NF-kB subunit	NF-kappa-B transcription factor complex
SERPING1	Serpin family G member 1	Regulation of the complement cascade
SF3B1	Splicing factor 3b subunit 1	Splicing factor, component of U2 snRNP
SREBF1	Sterol regulatory element binding transcription factor 1	Transcription factor for sterol regulatory element-1 (SRE1)
SRPK2	SRSF protein kinase 2	Splicing factor, protein serine/threonine kinase activity

### Subset for LoF

LoF genes were analyzed with the rationale that these are high-risk genes for schizophrenia (23). When the overall LoF database was compared with the IHR gene database, enrichment was found (n=452 or 28%; Chi-square p=0.0001), indicating that a large portion of IHR genes are potentially mutation-intolerant. The schizophrenia GWAS dataset as such was enriched with N=112 LoF genes (p=0.0001), as to be expected (23); thereby, n=25 IHR genes were among the LoF genes for schizophrenia, indicating enrichment (p=0.0002). When BH genes (30) were tested, n=35 genes were matched to LoF genes in schizophrenia (p=0.0001). Further, n=23 HDB genes were overlapping LoF genes in schizophrenia (p=0.0005). Taken together, a subset of schizophrenia genes can be defined by the combination of LoF and potential for change in expression levels during ischemic-hypoxic challenges.

### Subset for Monogenic Disorders

In a separate approach, GWAS-derived schizophrenia genes were selected if a mutation was known to affect development or function of the nervous system. This annotation lead to n=96 genes (21%) of the schizophrenia GWAS dataset linked to a monogenic disorder of the nervous system. Therein, genes from the IHR dataset (n=19; p=0.008) and BH dataset (n=23; p=0.002) were enriched in the subset of schizophrenia genes matching a monogenic disorder, whereas the HDB dataset showed only a trend (n=16; p=0.05).

### Subset for Synaptic Proteins

In view of multiple findings related to neurotransmission and synaptic functions in the literature for genomics of schizophrenia (25), synaptic genes within the schizophrenia GWAS dataset were analyzed in the next step. To do this, a gene/protein set curated for synaptic functions (n=1,112) was downloaded from SynGO (27). First, we tested whether synaptic genes are enriched among the IHR genes and found n=270 (24%; p=0.0001) shared genes which indicates that synaptic genes are strongly involved in the response to ischemia-hypoxia as such. For confirmation, a DAVID Bioinformatics analysis of the complete IHR gene set was run; the Gene Ontology (GO) term “synapse” (GO:0045202) was found highly enriched (p=2.2E-25, Bonferroni corrected p=1.22E-22). Next, we tested whether synaptic genes are enriched in the schizophrenia GWAS gene set as such and found n=46 shared genes (p=0.0095). Finally, when overlapping these n=46 schizophrenia genes listed in SynGO with IHR genes, n=20 were matched (p=0.0001); n=13 for BH genes (p=0.0064); and n=6 for HDB genes (p=0.6).

### Subset of Multiomics Data

Subsequently, additional gene sets derived from schizophrenia GWAS data were searched for confirmation, and the dataset of n=104 genes related to schizophrenia by multiomics studies (28) was selected. Enrichment was found for IHR genes (n=31; p=0.0001), but not for BH genes (n=27; p=0.03) or HDB genes (n=22; p=0.095).

### Neurodevelopmental Disorders

Furthermore, it was explored whether the role of IHR genes can be extended more broadly to neurodevelopment disorders, and a gene set for DD/ASD was analyzed (n=253) (29). Thereby, only 14/235 of the DD/ASD genes were overlapping with the schizophrenia GWAS dataset, indicating an independent dataset. Strong overlap was observed for the DD/ASD gene set with IHR genes (n=47; p=0.002), the BH dataset (n=72; p=0.0001), and HDB genes (n=59; p=0.0007).

### Exploration of HIFs

HIFs are major regulators of gene expression in response to hypoxia (36, 37). Therefore, an initial survey of GWAS-based schizophrenia genes for links to HIF regulation was performed using expert curation (38). Specific annotations for HIF-related genes in the schizophrenia GWAS dataset yielded n=33 genes (**Table 2**).

**Table 2:** Annotation of schizophrenia genes derived from genome-wide association study (GWAS) for a role of hypoxia-inducible factors (HIFs).

Gene symbol	Official full name	Main biological function
ALDOA	Aldolase, fructose-bisphosphate A	Glycolytic enzyme
ALPK3	Alpha kinase 3	Protein serine/threonine kinase
BNIP3L	BCL2 interacting protein 3 like	Pro-apoptotic factor within the Bcl-2 family
BTG1	BTG anti-proliferation factor 1	Regulator of cell growth and differentiation
CDK2AP1	Cyclin dependent kinase 2 associated protein 1	Role in cell-cycle and epigenetic regulation
CPEB1	Cytoplasmic polyadenylation element binding protein 1	Regulation of mRNA translation
CPT1C	Carnitine palmitoyltransferase 1C	Regulation of beta-oxidation
CREB3L1	cAMP responsive element binding protein 3 like 1	Transfactor activated by ER stress
CUL3	Cullin 3	Role in polyubiquitination
EP300	E1A binding protein p300	Histone acetyltransferase, regulation of transcription
ESRP2	Epithelial splicing regulatory protein 2	Splicing regulator
FGFR1	Fibroblast growth factor receptor 1	Growth factor signaling
FURIN	Furin, paired basic amino acid cleaving enzyme	Subtilisin-like proprotein convertase
GPR135	G protein-coupled receptor 135	Orphan receptor
HSPA9	Heat shock protein family A (Hsp70) member 9	Heat shock protein 70 gene family, mitochondrial
KAT5	Lysine acetyltransferase 5	Histone acetyl transferases, DNA repair
KDM4A	Lysine demethylase 4A	Trimethylation-specific demethylase, repressor
KMT5A	Lysine methyltransferase 5A	Protein-lysine N-methyltransferase, SETD8
LRP1	LDL receptor related protein 1	Low-density lipoprotein receptor
LSM1	LSM1 homolog, mRNA degradation associated	Pre-mRNA splicing, mediating U4/U6 snRNP formation
MAD1L1	Mitotic arrest deficient 1 like 1	Role in mitotic spindle-assembly checkpoint, cell cycle
NEK1	NIMA related kinase 1	Serine/threonine kinase, cell cycle
NMB	Neuromedin B	Bombesin-like family of neuropeptides
OGFOD2	2-oxoglutarate and iron dependent oxygenase domain containing 2	Oxidation-reduction process
OTUD7B	OTU deubiquitinase 7B	Deubiquitinase
PGM3	Phosphoglucomutase 3	Glycogen formation and utilization
PPP2R2A	Protein phosphatase 2 regulatory subunit Balpha	Negative control of cell growth and division
PRMT1	Protein arginine methyltransferase 1	Protein arginine N-methyltransferase
RALGAPA2	Ral GTPase activating protein catalytic alpha subunit 2	GTPase activator
RPTOR	Regulatory associated protein of MTOR complex 1	Interaction with mTOR kinase, negative regulator
SF3B1	Splicing factor 3b subunit 1	Splicing factor, component of U2 snRNP
TCF4	Transcription factor 4	Helix-loop-helix transcription factor
ZEB2	Zinc finger E-box binding homeobox 2	DNA-binding transcriptional repressor

## Discussion

**Figure 2:**
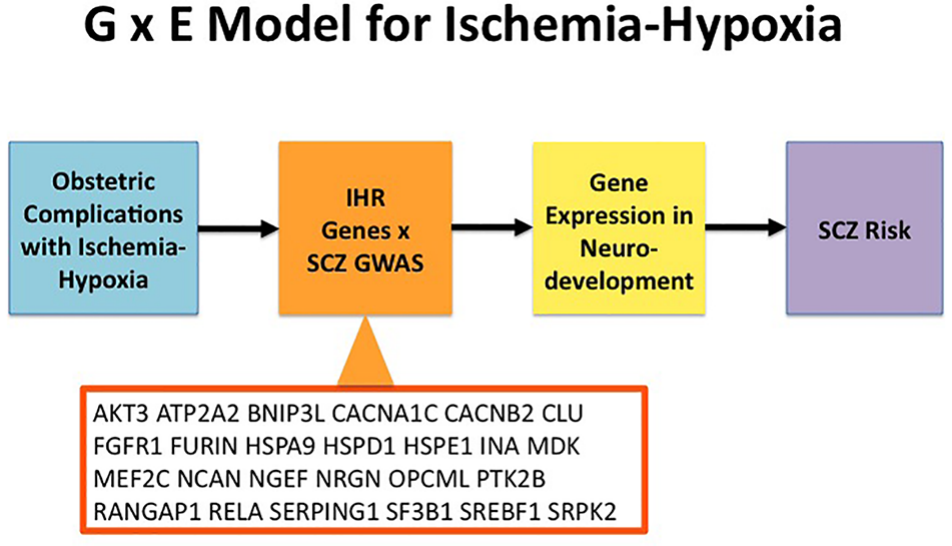
Model for Gene × Environment (G × E) interactions in relation to ischemia-hypoxia and Schizophrenia. Obstetric complications can cause ischemia-hypoxia that activates ischemia-hypoxia response (IHR) genes. A subset of genes from GWAS in schizophrenia (SCZ) overlap with IHR genes as shown here for the genes listed in **Table 1**. Subsequently, ischemia-hypoxia induces changes in gene expression for the overlapping SCZ genes during neurodevelopment. In turn, disturbances of neurodevelopment increase the risk of SCZ. GWAS, genome-wide association study; IHR, ischemia-hypoxia response; SCZ, schizophrenia.

Gene-environment interactions (G × E) play a key role in the neurodevelopmental model of schizophrenia. OCs are established environmental factors linked to the risk of schizophrenia which may involve ischemic-hypoxic events in the developing brain (5–7). The goal of this study was to apply datasets of experimentally defined genes responding to ischemia-hypoxia (IHR genes) to a large set of GWAS-defined schizophrenia genes. Thereby, the annotation for IHR genes opens the possibility that a matched schizophrenia gene is subject to regulation by ischemic-hypoxic periods occuring during OCs (i.e. during neurodevelopment). Genetic variation captured by GWAS may modify the ability of such genes to respond when challenged by ischemia-hypoxia; an oligogenic or polygenic abnormal response could negatively affect the developing brain. A graphic presentation of the G × E concept related to ischemia-hypoxia including the present analyses is provided in **Figure 2**.

While the overall gene set from the most recent GWAS (23) was not enriched for IHR genes, subsets of schizophrenia genes closely related to disease mechanisms were enriched based on a neurodevelopmental model. Genes from schizophrenia GWAS were enriched for mutation-intolerant genes (23), which in turn were strongly enriched for IHR genes. The enrichment of hypoxia-responsive genes among mutation-intolerant schizophrenia genes was replicated with two additional datasets (BH genes, HDB genes). A separate analysis showed that one fifth of genes from schizophrenia GWAS can be linked to monogenic disorders of the nervous system, which were also enriched for IHR genes and BH genes. We propose that the interaction between genetic variants for schizophrenia and effects of ischemia-hypoxia may affect the same cellular functions as the LoF or known mutations, although to a lesser degree. Thus, schizophrenia risk genes with a link to mutations affecting brain development and function provide a specific starting point for functional and experimental studies. In support, it was shown that a set of DD/ASD genes selected based on exome sequencing (29) was also enriched for IHR, BH and HBD genes.

Multiple pathway studies on genomic data in schizophrenia have converged on synaptic mechanisms (25, 26). Using a gene set for synaptic genes [SynGO; ref. (27)], enrichment was confirmed here for the genes in the GWAS loci for schizophrenia. In addition, it was shown with two independent methods that IHR genes are enriched in synaptic genes, indicating the involvement of the synaptic machinery in the response to brain ischemia-hypoxia. Subsequently, an enrichment for IHR and BH genes among the schizophrenia genes related to synaptic functions was detected. This subset of genes is proposed as suitable for functional *in vitro* studies of gene variants under hypoxic conditions.

A limitation of the present analysis is that IHR genes are a compilation from several experimental microarray studies of ischemia and hypoxia in animal models. Enrichments for three subsets of schizophrenia genes could be replicated with a large transcriptomics dataset derived by RNAseq in a focal brain ischemia model [“BH genes”; ref. (30)]. The analyses of enrichment for transcriptomics data was based on the assumptions that 18k genes are expressed in the developing brain and that the microarrays and RNAseq provided genome-wide coverage. Responses to ischemia and hypoxia mostly involve changes in transcription (“gene expression”) that have been described in multiple, independent experiments. Alterations of pre-mRNA splicing during ischemia-hypoxia also need to be considered (30). Since mRNA stability is also influenced by hypoxia (39), mRNA degradation may variably occur during early stages of ischemic neuronal damage. Genetic variants of schizophrenia risk genes may then influence gene expression, pre-mRNA splicing and/or mRNA stability after ischemia-hypoxia. Abnormal increases and decreases of the expressed proteins can alter neurodevelopmental trajectories, and impairment of protective responses can develop. A caveat is that a set of brain-expressed genes was tested against genomic data for a brain disorder; indeed, recent studies with gene sets obtained for different neuronal populations under physiological conditions showed enrichment with genomic data from schizophrenia (40). The dataset HDB was compiled across several organs on the basis of protein expression (31), and only the link for the subset of LoF genes was significant, but not for the subset for monogenic disorders or synaptic proteins. At this point, it remains unclear whether this finding was related to the use of protein studies which may be less sensitive than mRNA studies.

It remains possible that IHR genes are markers for highly responsive genes in the brain, because ischemia-hypoxia is a very strong stimulus for gene regulation; weaker stimuli such as abnormal neuronal activity could evoke regulation of the same genes that could be relevant for the pathophysiology of schizophrenia.

To address the issue of specificity, HIFs as major regulators of gene expression in response to hypoxia were analyzed (36, 37). The importance of low oxygen levels (relative hypoxia) and HIF regulation in neurodevelopment has been documented in great detail in experimental studies (38). Therefore, we here performed a survey of GWAS-based genes for links to HIF regulation and found several relevant genes, e.g. EP300 (11). Interestingly, a recent combined analysis of gene expression and GWAS data in schizophrenia showed a strong signal for HIF1A in the dorso-lateral prefrontal cortex (41). In the future, it will be of interest to see whether SNPs identified by GWAS in schizophrenia can be aligned with regulatory regions responding to HIFs.

A point of consideration is that our analysis has been focused on neurodevelopment under the assumption that gene x hypoxia interactions occur in the brain (9). A recent report has shifted the emphasis to gene expression in the placenta in relation to risk of schizophrenia (42). Evidently, the placenta is the central organ for oxygen supply to the embryo and fetus, and ischemic-hypoxic events of the brain during OCs often originate in placental dysfunction. The same gene variants related to ischemia-hypoxia will be expressed in the developing brain and placenta; therefore complex interactions can be envisioned that remain to be addressed. The novel observation that the schizophrenia GWAS dataset contains genes for monogenic disorders of neurodevelopment which overlap with IHR genes supports a role for brain mechanisms. Furthermore, overlap could be also shown for a set of DD/ASD genes which are specifically involved during neurodevelopment.

Vascular factors have been implicated in the emergence of schizophrenia during adolescence and early adulthood (12–14). Abnormalities in lactate levels and brain pH have been measured in manifest schizophrenia (43) which points to abnormalities in glycolytic pathways. Thus, a complementary view could be that the interaction between schizophrenia genes and ischemia-hypoxia (or closely related metabolic perturbations) occurs before or at the onset of psychosis during adolescence. However, direct evidence for vascular dysfunction or overt pathology of cerebral vessels in schizophrenia is presently not available. On the contrary, detailed stereological studies of the capillary system in chronic schizophrenia have not provided evidence for loss of microvessels (44), although larger vessels remain to be studied. It is also conceivable that episodic vascular events become important only on the background of a genetic susceptibility to impaired energy metabolism. In this respect, mitochondrial function has to be integrated into a late-onset model (45, 46).

Finally, the importance of gene variants may only become manifest under conditions of cellular stress (47). Thus, G × E designs using hypoxia are needed to put the hypothesis to the test for schizophrenia-related genes based on GWAS data. Studies capturing multiple medical conditions across the life-span, including detailed pre- and perinatal observations, are needed (48). Even then, the occurrence of ischemia-hypoxia in the fetal brain during OCs has to be assumed, based on experimental studies of maternal-fetal pathophysiology. Expression studies in animal models or cell culture of human neurons may provide new insight. In vivo neonatal models of ischemia-hypoxia have demonstrated regulation of some schizophrenia-related genes (49), but this approach does not capture variation associated with risk of schizophrenia. Studies on developing neurons derived from human iPSCs and exposed to variations in oxygen conditions may shed further light on functional effects of SNPs identified in GWAS for schizophrenia. Interestingly, an initial study using human iPSCs and CRISPR editing has explored a putative causal SNP in FURIN (50) which is an IHR gene and was captured by the present analysis of HIF targets.

A recent study looked for phenotype-specific enrichment of Mendelian disorder genes near GWAS loci across multiple complex traits (51). This provided the rationale for annotating genes in GWAS loci for monogenic (Mendelian) disorders of the nervous system that are known to affect development and function. A broad net was cast by searching for neurodevelopmental and neurological disorders, including disorders of the retina and hearing nerve. Many of these disorders may be traced back to effects of the mutations on early neural development. In the future, it can be tested whether SNPs found in GWAS studies of schizophrenia impact the same developmental process or neuronal function as a known mutation of the same gene.

In conclusion, the analysis of subsets of GWAS-defined schizophrenia genes related to putative disease mechanisms showed significant overlap with gene sets related ischemia-hypoxia, thereby providing cumulative evidence for a role of the hypoxia response in the etiopathogenesis of psychotic disorders. Further studies are warranted to define the genetic risk associated with ischemic-hypoxic events during OCs. Thereby, improved detection of abnormal blood flow and decreased oxygenation in the fetal brain is needed that could lead to novel treatment options.

## Data Availability Statement

The datasets generated for this study are available on request to the corresponding author.

## Ethics Statement

Ethics approval and written informed consent was not required as per local legislation and national guidelines.

## Author Contributions

RS-K wrote the first draft and all authors provided feedback. RS-K generated databases for ischemia-hypoxia response and carried out the enrichment analyses. TK provided expert annotations of HIF genes.

## Conflict of Interest

The authors declare that the research was conducted in the absence of any commercial or financial relationships that could be construed as a potential conflict of interest.
